# Ultra-Processed Food Intake, Obesity, and Mood Disorders: An Epidemiological Study From the National Health and Nutrition Examination Survey (NHANES) 2005 to 2018 Data

**DOI:** 10.7759/cureus.87975

**Published:** 2025-07-15

**Authors:** Kingsley O Ozojide, Victor U Chukwu, Damilola A Adeyemo, Omotola Akinade, Okelue E Okobi, Jeff G Lucien, Victoria Aliu

**Affiliations:** 1 Public Health, Nottingham Trent University, Nottingham, GBR; 2 College of Medicine, Abia State University, Uturu, NGA; 3 Research, Texas A&M University, Corpus Christi, USA; 4 Internal Medicine, General Hospital Ikorodu, Lagos, NGA; 5 Family Medicine, IMG Research Academy and Consulting LLC, Homestead, USA; 6 Family Medicine, Larkin Community Hospital Palm Springs Campus, Miami, USA; 7 Family Medicine, Lakeside Medical Center, Belle Glade, USA; 8 Pediatrics, Maimonides Medical Center, Brooklyn, USA; 9 Psychiatry, Ambrose Alli University/Irrua Specialist Teaching Hospital, Ekpoma, NGA

**Keywords:** body mass index (bmi), depression, dietary patterns, mental health, nhanes, obesity, ultra-processed food

## Abstract

Background: Ultra-processed food (UPF) consumption has become a prominent dietary pattern in modern populations, raising public health concerns due to its potential role in obesity and mental health disorders. This study examined the association between UPF intake, obesity, and depression among U.S. adults using nationally representative data.

Methods: Data were drawn from the National Health and Nutrition Examination Survey (NHANES) cycles 2005-2018, yielding a complete-case, weighted sample of 97,983,914 U.S. adults (n = 16,976). Survey-weighted means and proportions were estimated, and subgroup analyses were conducted by sociodemographic and health characteristics. Multivariable logistic and linear regression models assessed the relationships between UPF intake (as a percentage of energy intake) and obesity (BMI ≥ 30 kg/m²) and depression (Patient Health Questionnaire-9 (PHQ-9) score ≥ 10). All analyses incorporated NHANES's complex sampling design using STATA version 18 (StataCorp., College Station, TX, USA).

Results: The mean UPF intake was 22.36% (95% CI: 21.72-23.01). Higher UPF consumption was significantly associated with increased odds of depression (OR = 1.01; 95% CI: 1.00-1.01; p < 0.001), even after adjustment for age, weight, gender, physical activity, and race/ethnicity. However, no significant association was found between UPF intake and obesity (OR = 1.00; 95% CI: 0.99-1.00; p = 0.32). Linear regression results confirmed a positive association between UPF and PHQ-9 scores (β = 0.010; p < 0.001), but only a minimal relationship with BMI (β = 0.002; p < 0.05). Subgroup differences in UPF intake were observed by gender, physical activity, and mental health status.

Conclusion: Higher UPF intake is associated with increased depressive symptoms but not with obesity after controlling for confounding variables. These findings emphasize the potential mental health implications of ultra-processed dietary patterns and support public health strategies aimed at reducing UPF consumption to promote psychological well-being.

## Introduction

In the United States and other developed countries, people now rely heavily on ultra‑processed foods (UPFs). Since these products are made mainly from oils, fats, sugars, starches, and protein isolates, UPFs usually have additives that make them last longer and more appetizing [1]. Such foods as sugary drinks, packaged snacks, processed meat, instant food, and fast food are meant to be fast and easy for most people [2]. Because fast food is consumed so often, it has become a serious health concern in regard to obesity and mental health [3]. The NOVA classification system organizes foods by how much they are processed, and it now shows that UPFs account for more than 50% of the daily calories consumed by most U.S. adults [4,5].

In the United States, a significant public health concern is obesity since more than one in three individuals is considered obese. Evidence from different studies indicates an association between eating a lot of UPFs and increased body weight, which could be explained by these foods being less nutritious, having a high glycemic load, and being more attractive to eat [6,7]. Such foods usually contain many calories and are low in nutrients since they lack essential vitamins, minerals, and fiber, yet are packed with sugars, unhealthy fats, and simple carbohydrates [8]. The nutritional components of fast food are believed to interfere with energy use and hormonal balance, provoking an increase in body weight and the chances of diabetes, heart problems, and cancer [9]. Furthermore, because of their advanced engineering, UPFs can easily suppress our natural hunger, which may lead us to consume more calories and eat more of them [10].

More research suggests that what people eat can significantly influence their mental and physical health. Mental illnesses such as depression and anxiety are widespread and very concerning because they significantly reduce quality of life and add to the difficulties facing healthcare systems [11]. Studies indicate that when people eat many unprocessed foods, their mood and emotional health are not as good [12]. There are several proposed causes, including inflammation, increased stress on the body from free radicals, and changes in the gut-brain link; these could all become worse when UPFs are consumed [13,14]. After controlling for factors such as age, gender, race/ethnicity, and physical activity, researchers have seen a relationship between eating more UPFs and an increased risk of depression and anxiety [15]. However, a lot of these studies have been done on populations that are not representative of the whole population, and little research has looked at the role of UPF and obesity in affecting mental health [16].

Existing literature is limited, underscoring the need for large‐scale epidemiological studies to elucidate the relationships between UPF consumption, overweight status, and mood disorders. The present study uses National Health and Nutrition Examination Survey (NHANES) data to focus on diet and mental health because it includes dietary reports, physical check‑ups, and reliable mental health questionnaires from a wide range of individuals [17,18]. To understand the link between eating ultra‑processed foods and the rates of obesity and mood disorders, mainly depression and anxiety, among U.S. adults, NHANES data from 2005 to 2018 give the best insights [19-21]. The study’s main objective remains to investigate the associations between UPF consumption, depressive symptoms, and obesity in U.S. adults using data from the NHANES.

## Materials and methods

Study design and data source

This study employed a cross-sectional design using publicly available data from the NHANES, spanning seven consecutive two-year cycles from 2005 to 2018 [21]. The NHANES is conducted by the National Center for Health Statistics (NCHS) and utilizes a multistage, stratified, probability sampling method to assess the health and nutritional status of a representative sample of the non-institutionalized U.S. population. Data collection involved in-home interviews, standardized physical examinations, and 24-hour dietary recalls.

Study population

We included adult participants aged 18 years and older who completed the Day 1 dietary recall interview and participated in the physical examination component. Individuals who were pregnant or had missing data on key study variables, including dietary intake, body mass index (BMI), or depressive symptoms, were excluded from the analysis. The final analytic sample consisted of 16,979 participants.

Handling of missing data

Missing data were addressed using complete case analysis, given that the patterns of missingness were determined not to be missing completely at random. Instead of using imputation methods that may introduce bias under these conditions, we restricted the analytic dataset to individuals with complete information on all variables used in the analysis. As a result, observations with missing data on ultra-processed food intake, BMI, depression, or covariates were excluded before model estimation.

Dietary assessment and ultra-processed food classification

Dietary intake data were obtained from the Day 1 Individual Foods File (DR1IFF), which contains all foods and beverages reported by respondents in a 24-hour dietary recall interview. To classify food items by their level of processing, we applied the NOVA classification framework [22]. The NOVA system categorizes foods into four groups based on the extent and purpose of industrial processing: 1) unprocessed or minimally processed foods, 2) processed culinary ingredients, 3) processed foods, and 4) UPFs [23].

A food code-based NOVA classification lookup file was developed using the USDA Food and Nutrient Database for Dietary Studies (FNDDS). This file was merged with each NHANES dietary cycle using the USDA food code (dr1ifdcd). Items labeled under NOVA Group 4 were flagged as UPFs. Records with missing or unclassified food codes were excluded from UPF calculations.

We computed total daily energy intake (total_kcal) by summing the caloric content of all reported foods, Daily energy intake from UPFs (upf_kcal) by summing calories from Group 4 foods, Proportion of energy from UPFs (upf_pct) as: UPF% = (UPF kcal/Total kcal) × 100.

Outcome variables

Obesity

Anthropometric measurements were obtained during the physical examination. BMI was calculated as weight in kilograms divided by height in meters squared. Participants were classified as obese if their BMI was ≥ 30 kg/m². A binary variable, obesity (1 = obese, 0 = non-obese), was used in the analysis.

Depression

Depressive symptoms were measured using the Patient Health Questionnaire-9 (PHQ-9). A total score ranging from 0 to 27 was computed, with a cutoff of ≥10 used to define moderate to severe depression. A binary indicator, DEP, was created to represent depressive status (1 = depressed, 0 = not depressed).

Covariates

The study included several covariates selected based on theoretical relevance and prior literature. These were: age (continuous, in years), gender (male or female), race/ethnicity (as categorized by the NHANES), physical activity (based on self-reported vigorous work activity, coded as yes or no), and BMI (continuous).

Statistical analysis

All analyses were performed using Stata (StataCorp., College Station, TX, USA) and accounted for the complex, multistage sampling design of the NHANES. Survey weights (wtmec2yr), strata (sdmvstra), and primary sampling units (sdmvpsu) were combined and adjusted across the seven cycles (2005-2018) to create a 14-year dietary weight variable (wtmec7cycle), by NCHS guidelines. Weighted descriptive statistics were used to summarize participants' sociodemographic, dietary, and health characteristics. Means and proportions were reported for continuous and categorical variables, respectively, and UPF intake as a percentage of total energy was described across subgroups.

To assess unadjusted associations between UPF intake categories (quartiles or tertiles) and outcomes such as obesity and depression, we used weighted design-based F-tests for categorical variables. For adjusted associations, we estimated weighted logistic regression models for binary outcomes (obesity and depression) and linear regression models for continuous outcomes (BMI and PHQ-9 scores). All models controlled for age, gender, race/ethnicity, total energy intake, physical activity, and NHANES cycle.

Several sensitivity analyses were conducted, including reclassification of UPF intake into quartiles, adjustment for other dietary factors (e.g., total fat, fiber, food insecurity), exclusion of implausible energy intakes (<500 or >5,000 kcal/day), and stratification by age and gender. To examine subgroup differences, interaction terms were tested for UPF intake by gender, race/ethnicity, and physical activity level. Statistical significance was set at p < 0.05.

Ethical considerations

This study was based on secondary analysis of publicly available, de-identified data from the NHANES, administered by the U.S. Centers for Disease Control and Prevention (CDC). The NHANES protocols are reviewed and approved by the NCHS Research Ethics Review Board, and all participants provide written informed consent before data collection.

Given that our analysis did not involve direct contact with participants or identifiable information, this study was exempt from institutional review board (IRB) oversight under U.S. regulations for research involving secondary data.

## Results

Table 1 below presents the weighted sociodemographic, dietary, and health characteristics of NHANES participants from 2005 to 2018 (N = 16,976), representing a U.S. population size of approximately 97.98 million.

**Table 1:** Weighted sociodemographic, dietary, and health characteristics of NHANES participants (2005–2018), n = 16,976 This table presents the weighted sociodemographic, dietary, and health characteristics of the analytic sample (n = 16,976). Continuous variables are reported as sample‐weighted means (95% CI), and categorical variables as sample‐weighted percentages. Estimates account for the complex, multistage probability sampling design of the National Health and Nutrition Examination Survey (NHANES) using appropriate weights. Ultra-processed food (UPF) intake was calculated based on the NOVA classification using 24-hour dietary recall data. Depression status was defined by a Patient Health Questionnaire-9 (PHQ-9) score ≥10, and obesity was defined as BMI ≥30 kg/m². Physical activity reflects engagement in vigorous work-related activity. - : Intentionally left blank

Variable	Mean(95% CI) or N(%)
Age (years)	48.92(48.35-49.49)
Ultra-processed food (UPF)%	22.36(21.72-23.01)
Total kcal	1257.27(1237.71-1276.74)
Body Mass Index (kg/m^2^)	29.24(29.05-29.43)
Weight (kg)	83.06(82.49-83.64)
Height (cm)	168.34(168.08-168.61)
PHQ-9 Score	4.82(4.69-4.96)
Race/Ethnicity	-
Other Hispanic	7965566 (8.1%)
Non-Hispanic White	5461289 (5.6%)
Non-Hispanic Black	67346686(69%)
Non-Hispanic Asian	10592471(11%)
Other Race	6626990(6.8%)
Gender	-
Male	53358538(54%)
Female	97993002(46%)
Depression Status	-
Depressed (PHQ-9 score ≥ 10)	12906422(13%)
Non-depressed (PHQ-9 score < 10)	85086580(87%)
Obesity (BMI ≥ 30 kg/m²)	-
Obese	37773885(39%)
Non-obese	60219116(61%)
Physical activity (Vigorous work activity)	-
Yes	77563858(79%)
No	20420056(21%)

Table 1 above summarizes the weighted characteristics of the study population. The mean age of participants was 48.9 years (95% CI: 48.35-49.49). On average, UPFs contributed 22.4% (95% CI: 21.72-23.01) of total daily energy intake, while the mean total caloric intake was 1,257.3 kcal (95% CI: 1237.7-1276.7). The mean BMI was 29.2 kg/m² (95% CI: 29.05-29.43), indicating that, on average, participants were in the overweight range. Mean weight and height were 83.1 kg and 168.3 cm, respectively.

The mean PHQ-9 score was 4.8 (95% CI: 4.69-4.96), suggesting that most participants experienced minimal depressive symptoms. Based on the PHQ-9, 12906422 (13%) participants were classified as depressed (PHQ-9 ≥10), while 85086580 (87%) were not. The prevalence of obesity (BMI ≥30 kg/m²) was 37773885 (39%), while 60219116 (61%) of the population was non-obese.

In terms of gender, 53358538 (54%) of participants were male and 97993002 (46%) were female. Regarding race/ethnicity, the majority were Non-Hispanic Black 67346686 (69%), followed by Non-Hispanic Asian 10592471 (11%), Other Hispanic 7965566 (8.1%), Other Race (6.8%), and Non-Hispanic White 6626990 (5.6%). For physical activity, a majority, 77563858 (79%) reported engaging in vigorous work activity, while 20420056 (21%) did not.

Table 2 below shows the distribution of UPF intake across key demographic and health-related subgroups among NHANES participants (2005-2018).

**Table 2:** Distribution of UPF intake across key subgroups, n = 16,976 This table presents the weighted mean percentage of daily energy intake from ultra-processed food (UPF) across key demographic and health subgroups, based on National Health and Nutrition Examination Survey (NHANES) 2005–2018 data. Values are survey-weighted estimates with 95% confidence intervals (CI), reflecting the U.S. adult population. UPF intake was assessed using 24-hour dietary recall and categorized according to the NOVA classification system. Subgroups include categories for race/ethnicity, sex, depression status (Patient Health Questionnaire-9 (PHQ-9) score ≥10), obesity status (BMI ≥30 kg/m²), and engagement in vigorous work-related physical activity. All estimates account for the complex sampling design of the NHANES. - : Intentionally left blank

Variable	Mean UPF% % (95% CI)
Race/Ethnicity	-
Other Hispanic	22.11(21.16-23.06)
Non-Hispanic White	21.02(19.65-22.39)
Non-Hispanic Black	22.56(21.66-23.46)
Non-Hispanic Asian	23.99(22.94-25.04)
Other Race	19.21(17.55-20.87)
Gender	-
Male	22.10(21.28-22.94)
Female	22.68(21.89-23.47)
Depression Status	-
Depressed (PHQ-9 score ≥ 10)	25.10(23.66-26.55)
Non-depressed (PHQ-9 score < 10)	21.95(21.34-22.56)
Obesity (BMI ≥ 30 kg/m²)	-
Obese	22.74(21.95-23.52)
Non-obese	22.14(21.33-22.94)
Physical activity (Vigorous work activity)	-
Yes	21.59(20.94-22.24)
No	25.30(24.02-26.57)

From Table 2 above, it is evident that there are race/ethnicity differences in UPF consumption. The highest UPF intake was observed among Non-Hispanic Asians, with a mean of 24.0% (95% CI: 22.94-25.04), followed by Non-Hispanic Blacks at 22.6% (95% CI: 21.66-23.46), and Other Hispanics at 22.1% (95% CI: 21.16-23.06). Non-Hispanic Whites consumed slightly less UPF on average (21.0%, 95% CI: 19.65-22.39), while individuals from Other Races had the lowest intake (19.2%, 95% CI: 17.55-20.87).

Gender differences were minimal, with females reporting slightly higher UPF intake (22.7%, 95% CI: 21.89-23.47) than males (22.1%, 95% CI: 21.28-22.94). However, disparities were more pronounced when comparing depression status: participants classified as depressed (PHQ-9 ≥10) consumed a higher percentage of energy from UPFs (25.1%, 95% CI: 23.66-26.55) compared to those without depression (22.0%, 95% CI: 21.34-22.56).

Similarly, obese individuals (BMI ≥30 kg/m²) had higher UPF intake (22.7%, 95% CI: 21.95-23.52) than their non-obese counterparts (22.1%, 95% CI: 21.33-22.94). A notable difference was also observed by physical activity level: those who did not engage in vigorous work activity consumed more UPFs (25.3%, 95% CI: 24.02-26.57) compared to those who did (21.6%, 95% CI: 20.94-22.24). Overall, these findings suggest that higher UPF consumption may be associated with adverse health profiles and behaviors, including physical inactivity, obesity, and depression.

Table 3 below presents the weighted distribution of obesity, depression, and related health indicators across quartiles of UPF intake among NHANES participants from 2005 to 2018.

**Table 3:** Weighted distribution of obesity, depression, and related health indicators by quartiles of UPF intake, NHANES 2005–2018 (n = 16,976) Values are survey-weighted estimates from the National Health and Nutrition Examination Survey (NHANES) 2005–2018. Quartiles of ultra-processed food (UPF) intake were compared using design-based F-tests. Asterisks denote statistical significance: * p < 0.05, ** p < 0.01, *** p < 0.001 -: Intentionally left blank

Variable	Q1 (Lowest)	Q2	Q3	Q4 (Highest)	F-statistic	p-value
Obesity	-	-	-	-	1.63	0.18
Obese	23.69%	25.03%	25.98%	25.31%	-	-
Non-obese	25.53%	24.01%	25.07%	25.39%	-	-
Depression	-	-	-	-	7.53	p<0.001***
Depressed	22.56%	21.51%	26.1%	29.83%	-	-
Non-depressed	25.16%	24.84%	25.32%	24.68%	-	-
Body Mass Index (kg/m^2^)	29.05(28.78-29.33)	29.33(29.04-29.62)	29.30(29.00-29.61)	29.27(28.92-29.62)	-	-
Weight (kg)	82.47(81.59-83.36)	83.28(82.34-84.21)	83.47(82.48-84.47)	83.03(82.01-84.06)	-	-
Height (cm)	168.25(167.82-168.69)	168.25(167.82-168.68)	168.61(168.22-169.00)	168.26(167.83-168.69)	-	-
PHQ-9 Score	4.62(4.41-4.83)	4.64(4.46-4.83)	4.84(4.64-5.05)	5.17(4.95-5.40)	-	-

While obesity prevalence appeared to increase slightly across quartiles, rising from 23.7% in Q1 to 25.3% in Q4, the design-adjusted F-test revealed no statistically significant association between UPF intake and obesity status (F = 1.63, p = 0.18). This suggests that although obesity levels were marginally higher in the upper UPF quartiles, the differences were not statistically robust after accounting for the complex survey design.

In contrast, depression status showed a significant gradient across UPF quartiles. The proportion of participants with depression (PHQ-9 score ≥10) increased from 22.6% in the lowest UPF quartile to 29.8% in the highest (F = 7.53, p < 0.001). This trend supports a potential dose-response relationship between higher UPF consumption and elevated risk of depression, aligning with previous literature on diet and mental health.

For continuous health indicators, there were minor differences across quartiles. Mean BMI ranged narrowly from 29.05 kg/m² in Q1 to 29.27 kg/m² in Q4, and mean weight remained relatively stable across quartiles. Height did not vary meaningfully. However, PHQ-9 scores, reflecting depressive symptom severity, increased progressively with UPF intake, from 4.62 (95% CI: 4.41-4.83) in Q1 to 5.17 (95% CI: 4.95-5.40) in Q4. This pattern further reinforces the positive association between UPF intake and depressive symptoms.

Table 4 below presents the adjusted odds ratios (ORs) and 95% confidence intervals (CIs) from multivariable logistic regression models assessing the association between UPF intake and the odds of obesity and depression, after adjusting for relevant covariates.

**Table 4:** Multivariable logistic regression analysis of factors associated with obesity and depression (n = 16,976) Results are from multivariable logistic regression models assessing factors associated with obesity and depression, using survey-weighted National Health and Nutrition Examination Survey (NHANES) 2005–2018 data. Values represent adjusted odds ratios (ORs) with 95% confidence intervals (CI). Asterisks denote statistical significance: * p < 0.05, ** p < 0.01, *** p < 0.001 -: Intentionally left blank

	Obesity		Depression	
Variable	OR (95% CI)	p-value	OR (95% CI)	p-value
Ultra-processed food (UPF)%	1.00(0.99-1.00)	0.32	1.01(1.00-1.01)	p<0.001***
Age	1.02(1.02-1.03)	p<0.001	1.00(1.00-1.01)	0.078
Weight (kg)	1.18(1.17-1.19)	p<0.001	1.00(1.00-1.01)	0.002**
Female	2.17(1.91-2.48)	p<0.001	1.34(1.20-1.50)	p<0.001***
Vigorous work activity (No)	0.55(0.45-0.67)	p<0.001	0.97(0.85-1.10)	0.658
Other Hispanic	0.72(0.59-0.89)	0.003	1.40(1.12-1.76)	0.004**
Non-Hispanic White	0.26(0.23-0.30)	p<0.001	0.85(0.71-1.02)	0.073
Non-Hispanic Black	0.43(0.35-0.51)	p<0.001	1.13(0.93-1.38)	0.206
Non-Hispanic Asian	0.38(0.29-0.52)	p<0.001	0.93(0.75-1.16)	0.523

For obesity, UPF intake was not significantly associated with increased odds (OR = 1.00; 95% CI 0.99-1.00; p = 0.32). Although BMI is derived from both weight and height, we included weight as a covariate to account for absolute mass that may reflect body composition variations independent of height, acknowledging the inherent correlation with BMI. Significant predictors of obesity included age (OR = 1.02; 95% CI 1.02-1.03; p < 0.001) and female gender (OR = 2.17; 95% CI 1.91-2.48; p < 0.001). Engaging in vigorous work activity remained protective (OR = 0.55; 95% CI 0.45-0.67; p < 0.001).

With regard to depression, higher UPF intake was significantly associated with greater odds of depressive symptoms (OR = 1.01, 95% CI: 1.00-1.01, p < 0.001), even after adjusting for sociodemographic and lifestyle variables. This finding aligns with the hypothesis that dietary patterns rich in UPFs may adversely impact mental health. Other significant predictors included female gender (OR = 1.34, 95% CI: 1.20-1.50, p < 0.001) and weight (OR = 1.00, 95% CI: 1.00-1.01, p = 0.002), suggesting slightly elevated depression risk with increased body weight and among women.

Regarding race/ethnicity, notable differences emerged in both models. Compared to Other Hispanic participants, Non-Hispanic White (OR = 0.26, p < 0.001), Non-Hispanic Black (OR = 0.43, p < 0.001), and Non-Hispanic Asian (OR = 0.38, p < 0.001) groups had significantly lower odds of obesity. For depression, participants identifying as Non-Hispanic White had marginally lower odds compared to Other Hispanic, but the association did not reach conventional significance (p = 0.073). However, Non-Hispanic White ethnicity was significantly associated with increased odds of depression (OR = 1.40, p = 0.004) when compared with Other Hispanic.

Table 5 below displays results from two multivariable linear regression models evaluating the associations of UPF intake and selected covariates with continuous outcomes: BMI and PHQ-9 depression scores.

**Table 5:** Multivariable linear regression analysis of factors associated with BMI and depressive symptoms (PHQ-9 Score) (n = 16,976) Results are from multivariable linear regression models using survey-weighted National Health and Nutrition Examination Survey (NHANES) 2005–2018 data. Coefficients represent the estimated change in body mass index (BMI) (kg/m²) or Patient Health Questionnaire-9 (PHQ-9) depression score associated with each predictor, controlling for other covariates. Standard errors are shown in parentheses. Asterisks denote statistical significance: * p < 0.05, ** p < 0.01, *** p < 0.001 -: Intentionally left blank

Variable	Body Mass Index (kg/m^2^)	p-value	PHQ-9 Score	p-value
Ultra-processed food (UPF)%	0.002^*^	p< 0.05	0.010^***^	p< 0.001
-	(0.001)	-	(0.002)	-
Age (years)	0.035^***^	p< 0.001	0.008^**^	p< 0.01
-	(0.002)	-	(0.003)	-
Weight (kg)	0.293^***^	p< 0.001	0.009^***^	p< 0.001
-	(0.002)	-	(0.002)	-
Vigorous work activity (No)	-1.096^***^	p< 0.001	-0.026	-
-	(0.088)	-	(0.092)	-
Female	1.174^***^	p< 0.001	0.562^***^	p< 0.001
-	(0.062)	-	(0.099)	-
Other Hispanic	-0.437^***^	p< 0.001	0.728^**^	p< 0.01
-	(0.118)	-	(0.220)	-
Non-Hispanic White	-2.142^***^	p< 0.001	-0.327^*^	p< 0.05
-	(0.094)	-	(0.139)	-
Non-Hispanic Black	-1.501^***^	p< 0.001	0.253	-
-	(0.103)	-	(0.170)	-
Non-Hispanic Asian	-1.477^***^	p< 0.001	-0.042	-
-	(0.128)	-	(0.163)	-
Constant	4.643^***^	p< 0.001	3.325^***^	p< 0.001
-	(0.226)	-	(0.278)	-
Observations	16976	-	16976	-
R^2^	0.814	-	0.012	-

In the BMI model, UPF intake showed a positive but modest association with BMI (β = 0.002, p < 0.05), indicating that each percentage point increase in UPF consumption was associated with a 0.002-unit increase in BMI, after adjusting for other variables. Although statistically significant, the effect size is minimal, suggesting limited clinical relevance. In contrast, weight (β = 0.293, p < 0.001), female gender (β = 1.174, p < 0.001), and age (β = 0.035, p < 0.001) were strong, significant predictors of higher BMI. Engaging in vigorous work activity was significantly associated with lower BMI (β = -1.096, p < 0.001), suggesting a protective effect of physical activity. Additionally, several race/ethnicity groups, particularly Non-Hispanic White (β = -2.142, p < 0.001) and Non-Hispanic Black (β = -1.501, p < 0.001), had significantly lower BMI compared to the Other Hispanic reference group.

For the PHQ-9 depression score, UPF intake was again positively associated with depressive symptoms (β = 0.010, p < 0.001), indicating that greater UPF consumption correlates with higher depression scores. While the absolute effect was small, this association remained significant even after controlling for demographic and lifestyle factors, supporting a potential link between diet quality and mental health. Other key predictors of higher PHQ-9 scores included female gender (β = 0.562, p < 0.001), weight (β = 0.009, p < 0.001), and age (β = 0.008, p < 0.01). In contrast, vigorous physical activity was not significantly associated with depression (β = -0.026, p = 0.39). Regarding ethnicity, Non-Hispanic White participants reported lower PHQ-9 scores (β = -0.327, p < 0.05), while Other Race/Ethnicity groups, such as Other Hispanic (reference group) and Non-Hispanic Black, tended to report higher scores.

Notably, the BMI model explained a substantial proportion of variance (R² = 0.814), indicating strong model performance. However, the PHQ-9 model had a low explanatory power (R² = 0.012), suggesting that additional psychosocial, environmental, or clinical factors not included in the model may influence depressive symptoms.

## Discussion

This study utilized nationally representative data from NHANES 2005-2018 to investigate the associations between UPF intake, obesity, and depression among U.S. adults. By employing survey-weighted analyses and multivariable models, our findings contribute to the growing body of evidence linking dietary patterns, particularly UPF consumption, to adverse physical and mental health outcomes.

Consistent with existing literature, the average UPF intake accounted for over 22% of total caloric consumption, highlighting the pervasive nature of ultra-processed foods in the American diet [24-26]. Our subgroup analysis revealed that UPF intake was highest among individuals with depression and those who did not engage in vigorous physical activity, suggesting that lifestyle and psychosocial factors may influence dietary behaviors. Notably, UPF intake was also slightly higher among females and non-Hispanic Asians, though variability by race/ethnicity was observed. Consequently, the bivariate analysis did not find a statistically significant association between UPF quartiles and obesity prevalence (F = 1.63, p = 0.18); a clear gradient emerged in depression status, with the prevalence of depression rising across increasing UPF quartiles (F = 7.53, p < 0.001). This pattern was corroborated in multivariable logistic regression: higher UPF intake was significantly associated with increased odds of depression (OR: 1.01; 95% CI: 1.00-1.01; p < 0.001) after adjusting for confounders. However, no significant association was found between UPF intake and obesity in the adjusted model.

Linear regression results further supported these findings. A small but significant positive association was found between UPF consumption and PHQ-9 depression scores (β = 0.010, p < 0.001), while the association with BMI was minimal (β = 0.002, p < 0.05), suggesting that UPF intake may be more relevant to mental health than to obesity in this population. Other predictors of depression included female gender, greater weight, and certain racial/ethnic backgrounds, aligning with previous epidemiologic evidence on social and biological determinants of mood disorders. Interestingly, physical activity was inversely associated with obesity but not depression, underscoring the role of lifestyle behaviors in shaping metabolic outcomes. The strong association between higher body weight and both obesity and depression outcomes highlights the complex interplay between physiological and psychological health.

Comparatively, various studies have disclosed that UPF can cause neuroinflammation due to the imbalance in oxidant and anti-oxidant mechanisms, low levels of prebiotics and minerals, and elevated consumption of refined carbohydrates. Industrial packaging exposes UPF to phthalates and bisphenols, which induce inflammation by elevating biomarkers such as 1L-6, IL-10, and CRP [27]. Activation of glial cells, particularly microglia, is crucial in neuroinflammation, especially by inflammatory and cytotoxic mediators. These mediators include IL-1β, IL-6, tumor necrosis factor alpha (TNFα), IL-10, IL-18, IL-33, interferon-gamma (IFN-γ), β-amyloid, and lipopolysaccharide (LPS) [27,28]. This elicits a protective, regulated neuroinflammatory response in the brain, allowing it to adapt to stress without neuronal damage. Upon chronic exposure to the stressor, a sustained release of proinflammatory cytokines occurs, resulting in the continuous activation of glial cells. This culminates in an unregulated neuroinflammatory response in the brain, the generation of reactive oxygen species (ROS) that amplify inflammation, and damage to neural tissue. This effect disrupts neurotransmitter balance, resulting in a reduction in serotonin and dopamine, which are neurotransmitters associated with the pathophysiology of depression. It also results in hyperactivity of the hypothalamic-pituitary-adrenal (HPA) axis and increased levels of glutamate, which disrupts neuroplasticity, causing brain dysfunction and predisposing to depression [29].

Concerning the HPA axis, sustained stress and neuroinflammation disrupt the HPA axis, leading to hyperactivity through the downregulation of glucocorticoid receptors, impaired negative feedback mechanisms, and glucocorticoid resistance. The effect leads to a cycle of stress, an inflammatory response, neuronal damage, and disturbances in mood and cognition [29]. IL-6 is a significant cytokine that is elevated in depression and plays a role in influencing the HPA axis, neurotransmission of serotonin, and mood regulation. Oxidative stress and high ROS cause neuronal damage, leading to dysfunction in synaptic plasticity and neurogenesis by inhibiting the signal transduction network and progenitor cell proliferation. These effects are associated with symptoms seen in depression [29].

Proinflammatory cytokines also affect the kynurenine pathway, which is involved in tryptophan metabolism. These cytokine channels tryptophan metabolism to produce kynurenine, which crosses the blood-brain barrier to generate a high number of neurotoxic by-products, including kynurenic acid and quinolic acid, following metabolism by astrocytes and microglia. The increased kynurenic acid acts as an antagonist to neuronal synapses and blocks N-methyl-D-aspartate (NMDA) receptors, which inhibit neurotransmitter release, such as serotonin, dopamine, and glutamate. The effect causes cognitive dysfunction, and essentially, the blockade of serotonin is associated with depressive symptoms. On the other hand, increased quinolinic acid acts as an agonist to neuronal synapses and activates the NMDA receptors, which results in lipid oxidative degradation and excitotoxicity. These effects lead to neuronal damage, oxidative injury, impaired neuroplasticity, and depression [29,30].

Gut-brain axis and microbiota disruption in relation to UPF intake

Ultra-processed foods are known to contain large quantities of sugars, salts, saturated and trans fats, as well as additives that have various effects on the human body. The effects range from alterations in physiological processes, metabolic processes, cognitive function, and autoregulatory function of the human body, among others [25]. An observational cohort study by Varun et al. linked UPF consumption with adverse brain health outcomes, including increased risks of stroke and cognitive decline, even among individuals following otherwise healthy diets [26].

The enormous number of microbiota organisms in the gut system plays a vital role in human health, influencing responses to immunity, physiological processes, metabolic processes, and cognitive function [27,28]. Ultra-processed foods affect the homeostasis of the gut microorganisms by changing the diversity and composition of the gut microbiota, affecting the intestinal lining and predisposing to mental health disorders such as bipolar disorder and mood dysregulation. The gut-brain axis has a multidirectional communication system that enables it to communicate with each of the organ systems. When the immune system is compromised due to excessive ingestion of ultra-processed food, it produces neurotransmitters that enhance neuronal function [26]. The vagus nerve plays a crucial role in the gut-brain axis; its involvement in peristaltic movement, gastric emptying, enzyme secretion, and nutrient absorption cannot be overstated. Autoimmunity and dysregulation in the microbiota system influence the neuronal system due to their effect on the production and regulation of neurotransmitters such as gamma-aminobutyric acid (GABA), tryptophan, and serotonin, a precursor. This can lead to mood disorders, Parkinson’s disease, and multiple sclerosis, to mention a few [25-28]. Tryptophan is a precursor to serotonin, a major neurotransmitter that leads to the upregulation of indoleamine 2,3-dioxygenase, causing a diversion of tryptophan from serotonin synthesis towards the kynurenine pathway. Decreased serotonin levels increase neurotoxic metabolites, which promote depression and neurodegeneration. When immune function is compromised, the metabolism of specific metabolites and neurotransmitter precursors, many of which are derived from the microbiota, is disrupted.

Further, in addition to their lower nutritional value and effect on hormone balance, UPFs pose serious health risks, which stem from contamination during production and additives. With the increasing demand in the developed nations, there is a corresponding increase in production and the need for preservation. Food additives are used to add taste, color and flavor during processing, and increase the shelf life of UPFs [31,32]. However, research has identified a relationship between additives and allergies, asthma, headaches, vomiting, brain tumor, leukemia, cancer, damage to the liver and kidneys, attention deficit hyperactivity disorder (ADHD) and other mental health problems [31]. This relationship between food additives and ADHD has been repeatedly proven [33,34]. Moreover, it has also been proposed that benzo(a) pyrene [35] and lead [36], which are common contaminants during the production of UPFs, disrupt neurotransmitters and can cause anxiety and depression [37]. These findings highlight the potential for UPFs to contribute to a range of adverse health outcomes, underscoring the need for further investigation and public health attention.

Limitations

Despite its strengths, this study has some limitations. First, the cross-sectional design precludes any causal inference between UPF intake and the outcomes of interest. Second, although we used robust dietary classification through the NOVA system, some food items could not be categorized due to insufficient information, resulting in their exclusion. This reduction in total dietary representation may have underestimated the true UPF burden. Finally, self-reported measures, including dietary intake and physical activity, are susceptible to recall and social desirability biases. Future longitudinal studies are needed to clarify the temporal relationships between UPF consumption, obesity, and mental health outcomes. Additionally, refining dietary categorization frameworks to minimize unclassified items would enhance the precision and completeness of UPF exposure assessments. To strengthen the validity of our findings, we performed sensitivity and interaction analyses. These additional checks supported the robustness of the observed associations and did not materially alter the study conclusions.

## Conclusions

This study demonstrates that greater intake of ultra-processed foods is significantly associated with higher levels of depressive symptoms among U.S. adults, even after adjusting for key sociodemographic and lifestyle factors. However, no significant association was found between UPF consumption and obesity. These findings suggest that while UPF intake may not directly impact weight status, it could play a meaningful role in mental health, particularly depression. Public health efforts aimed at reducing UPF consumption may benefit from incorporating mental health promotion strategies to improve overall well-being in the population. Further, integrating dietary screening and counseling into the management of mood disorders may offer valuable insights and complementary support. Recognizing the potential impact of nutrition on mental health could enhance treatment approaches, fostering a more holistic perspective in patient care.
